# The less obvious effect of hosting the Olympics on sporting performance

**DOI:** 10.1038/s41598-022-27259-8

**Published:** 2023-02-02

**Authors:** Gergely Csurilla, Imre Fertő

**Affiliations:** 1 Institute of Economics, Centre for Economic and Regional Studies, Tóth Kálmán U. 4., 1097 Budapest, Hungary; 2 Sport Economics and Decision Making Research Centre, Hungarian University of Sports Science, Budapest, Hungary; 3 Institute of Agricultural and Food Economics, Hungarian University of Agriculture and Life Sciences, Gödöllő, Hungary; 4 Department of Economics, Czech University of Life Sciences, Prague, Czech Republic

**Keywords:** Environmental social sciences, Mathematics and computing

## Abstract

Hosting the Olympics has long been claimed to bring a financial return on investment. When this cannot clearly demonstrated, the medal surplus associated with host status is usually highlighted. We investigate the magnitude of having a home advantage at the recent Summer Olympic Games (1996–2021) separately for each organising country and by gender. Beside the host effect, the ex-ante and post impact on the medal surplus is also investigated in the same way. We analyse this effect using three models at the level of total medals, and medals won by men, and by women. Because of the number of zero observation in the response variable, we employ a zero-inflated negative binomial estimator. Our results cast some doubt on the claim of a host effect of the Olympics: when we control for socioeconomic variables in the models, the host effect fades away. Any host effect is clearer for medals won by men. Ex-ante and post effects on host countries are detectable in some cases but also fade with the inclusion of control variables. Based on our results, the medal surplus associated with the hosting of the Olympics is less evident than reported in literature or public discourse.

## Introduction

Hosting the Olympic Games is not clearly an economic success story. Bids for hosting the Olympic Games are mostly justified by economic impact studies which are strongly influenced by politicians^1^. As a consequence of the scandals related to the Olympics in past decades, the International Olympic Committee (IOC) introduced in 2014 the Agenda 2020 model to reform the bidding process for the Games^2^. One of the main objectives of Agenda 2020 was to make the organisation of the Olympics more economical and sustainable in response to the declining interest in bidding.

Another argument often made in favour of hosting the Olympics is the surplus in medal count due to the home advantage^3^. Numerous factors are attributed to having a home advantage. The cost of attending is minimized, athletes compete in facilities tailored to their needs, they are more motivated in front of a home crowd, and in judged events referees may decide in favour of the home athlete or team^4,5^. National and confirmation biases can also be observed in the judged events at the Olympic Games^6,7^. The Covid-19 behind closed doors experiments showed that the removal of social pressure reduces not only home advantage but also referee bias^8,9^. Partly due to the increase in governmental resources dedicated to sports ahead of a home Olympics, it is claimed that countries typically win an additional 1.8 percent of medals^4^.

**Figure 1:**
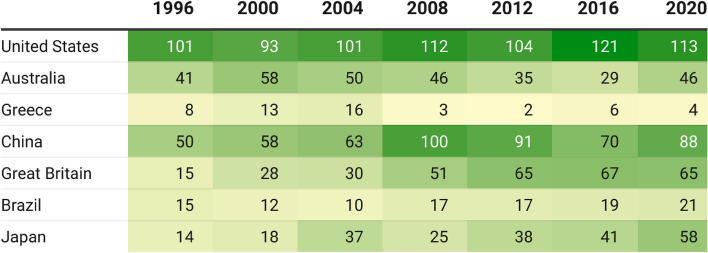
Medal count of host countries: Summer Olympic Games.

The host effect is not present in every sport^10^, and the significant medal surplus of host countries fades away immediately after hosting^11^. Moreover, a recent study highlights that the medal-related benefits of hosting the Olympics are diminishing^12^. An ex-post investigation may provide evidence about the Olympic performance of host countries compared to their previous medal count and account for socioeconomic conditions. As shown in Fig. 1, the medal surplus associated with the hosting is not so straightforward when visualized on a heat map. If countries have a noticeable tendency to win more medals at hosted Olympics, this trend should be more visible in the figure.

The aim of the paper is to investigate the magnitude of the home advantage at the recent Summer Olympic Games. Previous studies did not examine the medal surplus of hosts separately, our research would fill this gap in the literature. In addition to the home advantage, the ex-ante and post effect of hosting the Games is also analysed. We explore not only the host advantage based on total medals, but also present gender differences, similar to recent studies^12,13^. In some countries, women still do not have the same rights as men, and this is reflected in Olympic results^14^. There are also still gender gaps in the number of medals that can be won in many sports. These differences can also lead to differences in the impact of hosting. We use sport-level data to control the sport-level heterogeneity of a country. Sport-level datasets have rarely been used before in the literature^12^. Following Singleton et al. we also apply two-way fixed effects in the regression models to control for the heterogeneity in country performance in terms of number of medals at the Olympic Games. We control for GDP per capita (at purchasing power parity) and population, the two most important determinants of Olympic success^4,15–18^.

Our results cast some doubt on the presence of the host effect of the Olympics. Countries still use the narrative of the host effect to justify bidding for the Games. However, we provide evidence that in only a few of the last seven Summer Olympics can such an effect be identified. If there is no economic benefit of hosting the Olympics, or extra medals, why are the games worth bidding for?

Literature has typically focused on the impact of host-country status on the medal count in general. However, we are interested in the host effect on medal growth for each host country. The novelty of the approach is thus that we examine the medal-related benefit of hosting the Olympic Games on an individual country level and pinpoint the volatility of the effect.

## Conceptual background

Studies that investigate the determinants of sporting success usually apply a variable to control for the outstanding performance of the hosting country. Hosting is considered to provide tremendous benefits for the home team or athletes by conferring enormous benefits due to the support of spectators, familiarity with local conditions, and the intensity of public expectations^19^.

In football, the host effect is usually also associated with football tradition or culture^19–21^. Therefore, not only is the current host country included in the variable but also other former host countries. The results of studies on the host effect are contradictory. Some studies have found a significant performance increase for the host country^20,21^. However, when another proxy that captured football culture was employed in addition to the host dummy variable the effect lost its statistical significance^19^.

The case is completely different for the Olympics. Governments mobilize resources long before the host Olympic Games to help their athletes achieve outstanding results on the home field. An effect of hosting is usually employed in the related models to control for the outstanding medal surplus of the home country. The host effect clearly exists when using aggregated data; the host dummy variable shows a significant positive effect on the number of medals in all studies^4,5,11,12,15–18,22,23^.

However, there are clear differences when disaggregated data are employed. When individual and team sports are distinguished, the host effect is detectable only in the case of individual, not team sports^16^. For the Winter Olympic Games, the magnitude of the host effect is less than for the Summer Olympic Games, which may be explained by the much more restricted sample of competitors^17^.

The host effect cannot be observed in all sports—it is heterogeneous across sports. At the Summer Olympic Games, when sports were investigated individually, the effect of hosting was found to be statistically significant in only six of fifteen sports^10^. In subjectively judged events, an official may decide in favour of the home team or athlete^5,10,17,24^. However, a home bias is not detectable in all judged sports; in skating, no host advantage exists^17^.

The host effect also shows differences by gender. Leeds and Leeds find that the host effect can be detected for the gold medal in both genders, but in the case of silver and bronze medals only for men. However, a newer study shows a similar positive home advantage for both genders^5^. Gender differences between the Summer and Winter Olympics also exist, with the host effect for women being largely insignificant at the Winter Olympic Games^12,17^.

Last, a distinction can be made in terms of medal types in relation to the host effect. The host effect usually leads to proportionately more gains in gold medals than bronze or silver^23^.

The host effect is not only investigated in relation to the current host but also to previous and following hosting countries. Countries are chosen to host the Olympic Games at least seven years in advance. The underlying assumption is that the government of the host country may mobilize resources—e.g. allocate a larger budget for elite athlete programmes, or hire prominent foreign coaches—way before the hosted event^5,18,25^. Consequently, the earlier, more conscious preparation of athletes may be detectable in terms of medal surplus at the Olympics prior to the hosted one. Studies find a positive and strongly significant coefficient for the ex-ante host variable^18,25^. Nevertheless, a latter study omitted the ex-ante variable from their analysis, highlighting the overly short preparation time for developing a whole new sports strategy and the negligible effect compared to the host variable^5^.

A few studies have employed an ex-host dummy for previous host countries. It is assumed that countries that hosted the Games before have an advantage in terms of mid-term and long-term effects^26^. The former lasting beneficial effect is attributed to the lower cost and better conditions for Olympic preparation; the latter is due to better sporting systems^18,26^. Interestingly, only one study found a positive and significant correlation with medal totals^26^, and only one presents evidence of the immediate loss of the effect after hosting^11^, while the results of other papers are contradictory^16–18^. Nevertheless, a recent study finds evidence that countries have significantly improved their performance at both the previous and following Olympics when hosting the Summer Olympic Games^12^.

To summarize, the host effect has not been investigated before one by one at the level of the Olympics. The effect at the Olympic Games is clearly detectable if aggregate data level (country—Olympics) is used. The more the dataset is disaggregated, the more uncertain the evidence of host effect. The host effect is mostly present in judged sports, mainly in men’s events, and has the biggest impact on gold medals. The pre- and post-host dummies show some positive effects on the medal totals, but results do not offer clear evidence. Consequently, the hosting, pre- and post-hosting effect are worth investigating by creating separate dummy variables for each host.

## Data and methods

### Econometric model

We used the Olympic medal counts of countries for sports (total, men, and women) as the outcome variable. To measure the host effect separately for each Olympiad, we created dummy variables for each host country. For controlling countries average medal winning we employed a variable about the mean of the medals won by a country in the sample at an Olympic Game. Average medal winning is essential to get a reliable estimate of the hosting dummies. Using a lagged dependent variable as a control for previous performance tends to lead to bias if a fixed effect is employed in the model^27^. As the number of events and the medals that are obtainable change, we included two-way, Olympic Games, and sport-level fixed effects in the regression models to address the differences^12^. Due to the different model specification, in contrast to the country-sport and Game-level fixed effects used by Singleton et al., we employ sport- and Game-level fixed effects in our models. The baseline specification is the following:

1 $${m}_{i,j,t}=\mathrm{\alpha }+{\upbeta }_{1}{d}_{i,t}+{\upbeta }_{2}{AM}_{i,j}+{\uptheta }_{t}+{\mathrm{\varphi }}_{j}+{\upvarepsilon }_{i,j,t},$$

where ${m}_{i,j,t}$ is the medal count of country *i* in *j* sport at the *t* Olympic Games, ${d}_{i,t}$ is the dummy variable of host country *i* of *t* Olympics, and ${AM}_{i,j}$.is the average Olympic medal winning in the sample of country *i* in *j* sport. ${\theta }_{t}$ denotes the Games and ${\varphi }_{j}$ the sports-level fixed effect. ${\upvarepsilon }_{i,j,t}$ is the disturbance term, which we assume to be heteroskedastic.

There are two factors that determine a country’s success at the Olympics: economic resources, and the pool of talent^4,10,11,16,17^. We employ the logarithmic forms of GDP per capita and the size of population in the extended model to control for these factors. The extended specification is the following:

2 $${m}_{i,j,t}=\mathrm{\alpha }+{\upbeta }_{1}{d}_{i,t}+{\upbeta }_{2}{AM}_{i,j}+{\upbeta }_{3}{\mathrm{ln}GDPpc}_{i,t}+{\upbeta }_{4}{\mathrm{ln}POP}_{i,t}+{\upbeta }_{5}{Communist\, bloc}_{i}+{\uptheta }_{t}+{\mathrm{\varphi }}_{j}+{\upvarepsilon }_{i,j,t},$$

where ${\mathrm{ln}GDPpc}_{i,t}$, ${lnPOP}_{i,t}$ are the main control variables, the GDP per capita and population size of country *i* in year *t*. We use the logarithmic form of both variables. We also employed a dummy for countries that were member states of the Soviet Union or were under its influence. Previous studies have shown that these countries outperform their socio-economic counterparts in terms of Olympic medals even after the break-up of the Soviet Union^4,10,17,22^. The ${Communist \,bloc}_{i}$ variable indicates that country *i* was included in this group.

To detect ex-ante and post effects of Olympics hosting countries, model (1) and (2) were extended with pre- and post-dummies in the same way as with the host dummy. This model specification enabled us to distinguish previous and later effects from the medal surplus obtained from hosting.

**Figure 2:**
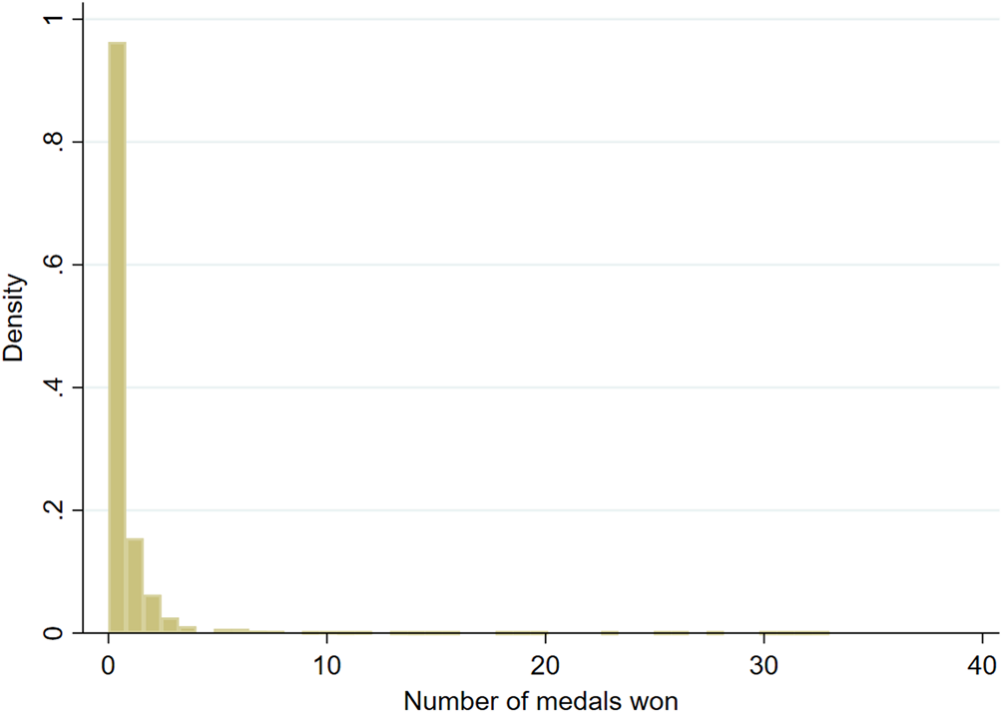
Distribution of total number of medals.

Since most countries do not win any medals at the Olympics, there are a lot of zero observations in the sample (see Fig. 2), which may bias the estimation using OLS. Previous studies used the Tobit estimator^4,5,10,11,16–18^ or zero-inflated beta regression^15^ to manage the zero-observation problem. However, medal count can only be a positive number, which indicates a Poisson or negative binomial distribution^28^. To account for both issues, zero-inflated Poisson (ZIP) or zero-inflated negative binomial (ZINB) models should be used^29^.

To differentiate the ZINB from the ZIP model, the negative binomial overdispersion parameter, α, can be used. In the case of the Poisson, the variance is equal to its mean; in contrast, there is overdispersion for the negative binomial, thus the variance is greater than the mean. Overdispersion is indicated if α > 0; moreover, the larger the α, the greater the negative binomial variance^29^. We undertook post-estimation tests for the different estimators. First, a likelihood-ratio test to compare ZINB with ZIP. Second, a Vuong test to compare ZINB with a standard negative binomial model.

### Data

We employed data about the medal counts of Summer Olympic Games between 1996 and 2021. Due to regime changes in the 1990s and earlier boycotts, the results of earlier Olympics cannot be used for a longitudinal analysis. Instead of aggregate data, we used the sport-level medal count to obtain more detailed information about countries’ Olympic performance^12,17^. The individual units are countries associated with specific sports (e.g., Afghanistan—athletics), and the time dimension is the year of the Olympic Games in the dataset. We used data for countries with qualified athletes in the sport concerned. Some countries have only participated in one Olympic competition, so using zero observations from all Olympic competitions would bias the analysis. For the socioeconomic indicators, we used data from the database of the World Bank^30^. The Olympic Games are a quadrennial event, so to obtain more detailed information on the economic and social situation in each country four-year geometric means were calculated for the year of the Olympic Games and the three years preceding it. This method eliminates bias due to data volatility or erroneous data. Summary statistics for the variables included in the analysis are presented in Table A1 in the Appendices.

## Results

We present our estimations using Eqs. (1) and (2) with three different outcome variables: medals won by total, men, and women. We employ in the models (1–3) with Eq. (1) the dummy variables and the average success as a control variable. The models (4–6) estimated based on Eq. (2) includes the usually used control variables (GDP, population, and communist past) in addition to the variables in the first equation.

First, to demonstrate that the host effect is detectable for the countries in the sample, we also estimated it with an aggregate variable (Table 1). All coefficients are significant, except for female events, at the 1% significance level. The effect of control variables decreases the strength of the host effect for women, while the explanatory power remains robust for men.

**Table 1:** Effects of hosting the Olympic Games with an aggregate dummy variable.

	Baseline model			Extended model		
	Total	Men	Women	Total	Men	Women
	(1)	(2)	(3)	(4)	(5)	(6)
**Main**						
Host	0.467***	0.473***	0.415***	0.257***	0.279***	0.198*
	(0.0820)	(0.103)	(0.113)	(0.0818)	(0.103)	(0.115)
AM	0.725***	1.222***	1.271***	0.600***	1.053***	1.108***
	(0.0295)	(0.0499)	(0.0642)	(0.0258)	(0.0509)	(0.0567)
lnGDPpc				0.415***	0.353***	0.464***
				(0.0237)	(0.0246)	(0.0314)
lnPOP				0.170***	0.126***	0.218***
				(0.0150)	(0.0182)	(0.0217)
Communist bloc				0.668***	0.584***	0.724***
				(0.0498)	(0.0580)	(0.0710)
OG FE	Yes	Yes	Yes	Yes	Yes	Yes
Sport FE	Yes	Yes	Yes	Yes	Yes	Yes
Constant	− 1.890***	− 2.348***	− 2.803***	− 8.878***	− 7.970***	− 11.14***
	(0.134)	(0.154)	(0.204)	(0.422)	(0.475)	(0.586)
**Inflate**						
lnGDPpc	− 1.232***	− 1.310***	− 1.253***	− 0.197	0.460	− 1.026***
	(0.114)	(0.185)	(0.150)	(0.622)	(1.017)	(0.377)
lnPOP	− 1.181***	− 1.433***	− 1.244***	− 1.312***	− 1.526***	− 1.358***
	(0.0976)	(0.187)	(0.124)	(0.138)	(0.218)	(0.230)
Constant	27.63***	31.16***	29.84***	18.42**	14.46	27.54***
	(2.024)	(3.509)	(2.674)	(7.270)	(9.509)	(6.202)
Observations	12,957	12,957	12,957	12,957	12,957	12,957

Robust standard errors in parentheses.

*p < 0.1, **p < 0.05, ***p < 0.01.

Then we examined the surplus of winning medals per hosts with separate dummy variables (Table 2). In the baseline specification (1–3), five from seven Olympic hosts were able to increase their medal counts significantly in all cases. Without control variables, the United States (1996), Australia (2000), China (2008), Great Britain (2012), and Japan (2021) clearly benefited from a home advantage in terms of medal total. United States (1996), Great Britain (2012), Brazil (2016), and Japan (2021) achieved a significant increase in medals won by men. Australia (2000), China (2008), and Great Britain (2012) were the only who won significantly more medals in women’s events when they hosted the Olympic Games. Greece (2004) was the only country that could not benefit from the advantages of the home field in either model specification.

**Table 2:** Effects of hosting the Olympic Games by host.

	Baseline model			Extended model		
	Total	Men	Women	Total	Men	Women
	(1)	(2)	(3)	(4)	(5)	(6)
OG96	0.508***	0.544**	0.149	− 0.00180	0.0872	− 0.430
	(0.190)	(0.277)	(0.386)	(0.195)	(0.279)	(0.392)
OG00	0.445**	0.277	0.621***	0.349**	0.161	0.553**
	(0.181)	(0.267)	(0.219)	(0.176)	(0.252)	(0.215)
OG04	0.273	0.316	0.302	0.380	0.362	0.512
	(0.262)	(0.263)	(0.410)	(0.254)	(0.263)	(0.399)
OG08	0.454**	0.298	0.697***	0.138	0.0615	0.349
	(0.207)	(0.309)	(0.242)	(0.184)	(0.273)	(0.233)
OG12	0.727***	0.649***	0.554**	0.562***	0.516**	0.376*
	(0.194)	(0.219)	(0.239)	(0.187)	(0.212)	(0.227)
OG16	0.259	0.701**	− 0.203	0.330	0.748***	− 0.103
	(0.242)	(0.277)	(0.432)	(0.246)	(0.287)	(0.435)
OG20	0.479**	0.439*	0.336	0.100	0.100	− 0.0424
	(0.243)	(0.254)	(0.329)	(0.229)	(0.237)	(0.302)
AM	0.724***	1.221***	1.264***	0.599***	1.052***	1.105***
	(0.0296)	(0.0500)	(0.0651)	(0.0258)	(0.0511)	(0.0567)
lnGDPpc				0.417***	0.356***	0.467***
				(0.0238)	(0.0248)	(0.0316)
lnPOP				0.174***	0.128***	0.223***
				(0.0154)	(0.0188)	(0.0220)
Communist bloc				0.673***	0.594***	0.719***
				(0.0501)	(0.0584)	(0.0713)
OG FE	Yes	Yes	Yes	Yes	Yes	Yes
Sport FE	Yes	Yes	Yes	Yes	Yes	Yes
Constant	− 1.890***	− 2.349***	− 2.786***	− 8.943***	− 8.022***	− 11.23***
	(0.134)	(0.155)	(0.204)	(0.426)	(0.481)	(0.586)
**Inflate**						
lnGDPpc	− 1.232***	− 1.312***	− 1.248***	− 0.187	0.486	− 1.032***
	(0.114)	(0.185)	(0.149)	(0.640)	(1.051)	(0.388)
lnPOP	− 1.180***	− 1.433***	− 1.240***	− 1.312***	− 1.530***	− 1.361***
	(0.0974)	(0.187)	(0.123)	(0.138)	(0.229)	(0.238)
Constant	27.62***	31.17***	29.75***	18.28**	14.21	27.57***
	(2.019)	(3.507)	(2.649)	(7.442)	(9.660)	(6.433)
Observations	12,957	12,957	12,957	12,957	12,957	12,957

Robust standard errors in parentheses.

*p < 0.1, **p < 0.05, ***p < 0.01.

When we added the control variables into the models (4–6), most of the significant results vanished. In terms of medal totals, only Australia and Great Britain remained significant. Great Britain and Brazil won significantly more medals in men’s sports, and Australia and Great Britain in women’s sports when we controlled for GDP per capita and population. For Greece, results did not change with the extended specification. The results do not present any home advantages for Greece in terms of medal won. The control variables changed the results of China, the medal surplus is not detectable in total and women’s medals anymore. Brazil similarly won significantly more men’s medals in 2016 when accounting for socioeconomic background. The rest of the countries do not present evidence for home advantage in none of the medal types when socioeconomic control variables—the most used in the literature—are employed in the models.

The results of ex-ante and post effects of hosting countries are presented in Table 3 in the same structure as in Table 2.

**Table 3:** Ex-ante and post effects on Olympic host countries.

	Baseline model			Extended model		
	Total	Men	Women	Total	Men	Women
	(1)	(2)	(3)	(4)	(5)	(6)
**Main**						
OG96	0.544***	0.566**	0.208	0.0207	0.102	− 0.390
	(0.191)	(0.277)	(0.389)	(0.196)	(0.280)	(0.395)
OG00	0.476***	0.294	0.646***	0.361**	0.165	0.555**
	(0.182)	(0.267)	(0.219)	(0.176)	(0.253)	(0.217)
OG04	0.273	0.311	0.319	0.384	0.360	0.531
	(0.262)	(0.263)	(0.411)	(0.254)	(0.264)	(0.400)
OG08	0.459**	0.291	0.737***	0.101	0.0203	0.335
	(0.206)	(0.308)	(0.241)	(0.185)	(0.274)	(0.237)
OG12	0.759***	0.694***	0.601**	0.577***	0.546**	0.389*
	(0.195)	(0.220)	(0.239)	(0.188)	(0.214)	(0.228)
OG16	0.310	0.767***	− 0.167	0.362	0.801***	− 0.0877
	(0.242)	(0.278)	(0.433)	(0.247)	(0.288)	(0.437)
OG20	0.496**	0.457*	0.364	0.114	0.119	− 0.0241
	(0.242)	(0.253)	(0.326)	(0.228)	(0.238)	(0.302)
Pre96	0.244	− 0.0614	0.610**	0.181	− 0.132	0.552*
	(0.235)	(0.377)	(0.284)	(0.225)	(0.346)	(0.283)
Pre00	0.347	0.454	0.235	0.446	0.500	0.431
	(0.332)	(0.324)	(0.428)	(0.331)	(0.328)	(0.419)
Pre04	− 0.295	− 0.723*	0.00501	− 0.454*	− 0.761**	− 0.199
	(0.295)	(0.438)	(0.238)	(0.246)	(0.352)	(0.227)
Pre08	0.205	− 0.184	0.784**	0.0678	− 0.265	0.551*
	(0.256)	(0.246)	(0.317)	(0.234)	(0.229)	(0.310)
Pre12	0.331	0.597**	0.296	0.387	0.630**	0.360
	(0.247)	(0.247)	(0.377)	(0.252)	(0.254)	(0.382)
Pre16	0.306	0.435	− 0.000362	− 0.100	0.0910	− 0.378
	(0.307)	(0.328)	(0.429)	(0.289)	(0.297)	(0.399)
Pre20	0.00174	− 0.0542	0.0951	− 0.169	− 0.199	− 0.106
	(0.198)	(0.224)	(0.274)	(0.190)	(0.213)	(0.279)
Post96	0.626**	0.754**	0.638	0.499*	0.604*	0.551
	(0.282)	(0.329)	(0.460)	(0.280)	(0.325)	(0.463)
Post00	0.375*	0.0839	0.273	− 0.189	− 0.411	− 0.359
	(0.208)	(0.323)	(0.343)	(0.212)	(0.327)	(0.350)
Post04	0.301	0.383	0.434	0.233	0.275	0.386
	(0.268)	(0.319)	(0.295)	(0.272)	(0.310)	(0.307)
Post08	− 0.953*	− 0.821	− 1.139	− 0.886*	− 0.805	− 0.971
	(0.512)	(0.658)	(0.987)	(0.516)	(0.654)	(1.002)
Post12	0.323	0.499	0.441**	− 0.139	0.0635	− 0.0952
	(0.232)	(0.355)	(0.222)	(0.213)	(0.328)	(0.219)
Post16	0.975***	1.121***	0.745***	0.778***	0.950***	0.532**
	(0.196)	(0.236)	(0.229)	(0.192)	(0.235)	(0.221)
Post20	0.375*	0.454*	0.593*	0.464**	0.546**	0.692**
	(0.208)	(0.261)	(0.347)	(0.204)	(0.252)	(0.349)
AM	0.716***	1.212***	1.249***	0.596***	1.049***	1.103***
	(0.0296)	(0.0507)	(0.0666)	(0.0258)	(0.0518)	(0.0569)
lnGDPpc				0.416***	0.355***	0.468***
				(0.0243)	(0.0253)	(0.0323)
lnPOP				0.178***	0.130***	0.227***
				(0.0162)	(0.0198)	(0.0231)
Communist bloc				0.696***	0.616***	0.743***
				(0.0508)	(0.0593)	(0.0722)
OG FE	Yes	Yes	Yes	Yes	Yes	Yes
Sport FE	Yes	Yes	Yes	Yes	Yes	Yes
Constant	− 1.911***	− 2.364***	− 2.821***	− 9.016***	− 8.062***	− 11.35***
	(0.134)	(0.156)	(0.206)	(0.444)	(0.500)	(0.608)
**Inflate**						
lnGDPpc	− 1.244***	− 1.323***	− 1.272***	− 0.209	0.501	− 1.060***
	(0.115)	(0.187)	(0.154)	(0.671)	(1.140)	(0.391)
lnPOP	− 1.190***	− 1.443***	− 1.259***	− 1.319***	− 1.542***	− 1.371***
	(0.0984)	(0.190)	(0.127)	(0.140)	(0.250)	(0.247)
Constant	27.85***	31.38***	30.19***	18.51**	14.16	27.89***
	(2.041)	(3.541)	(2.764)	(7.788)	(10.17)	(6.610)
Observations	12,957	12,957	12,957	12,957	12,957	12,957

Robust standard errors in parentheses.

*p < 0.1, **p < 0.05, ***p < 0.01.

Without the control variables, none of the countries showed significant gains in terms of the medal total at the Olympic Games before the hosting. In addition, China (Pre04) won significantly less medals in men’s events in 2004. Brazil was the only one who won more medals in men’s events in 2012 before the hosting. Australia and Great Britain were the sole countries to earn a significant number of additional medals in the Olympic Games prior to hosting but they win significantly more medals only in women’s events. Adding the control variables into the models, China’s significantly worse performance was also detectable in terms of medal totals. Rest of the effects remained the same.

The results are slightly better for the post effects compared to the pre effects. Four of the seven host countries in the sample were able to maintain or further increase their outstanding performance after the home event.

Spain (Post96), United States (Post00), Great Britain (Post16), and Brazil (Post20) won significantly more medals in total after hosting. However, Greece’s performance dropped significantly in terms of medal totals. While Great Britain and Brazil were also able to maintain the medal surplus both in men’s and women’s events, Spain could only win significantly more medals in the men’s events alone. China (Post12) also managed to gain a significant medal surplus in the women’s events.

Interestingly, Greece (Post08) won significantly fewer medals. As expected, the significant results slightly changed in the models extended with the control variables. Spain lost significance in terms of total and men’s medals, but the effects were still significant at 10 percent. Great Britain and Brazil remained significant in relation to almost every type of medal. The Chinese female performance surplus has diminished. With and without controlling for the socioeconomic situation, none of the other countries performed significantly better after hosting the Olympic Games.

**Figure 3:**
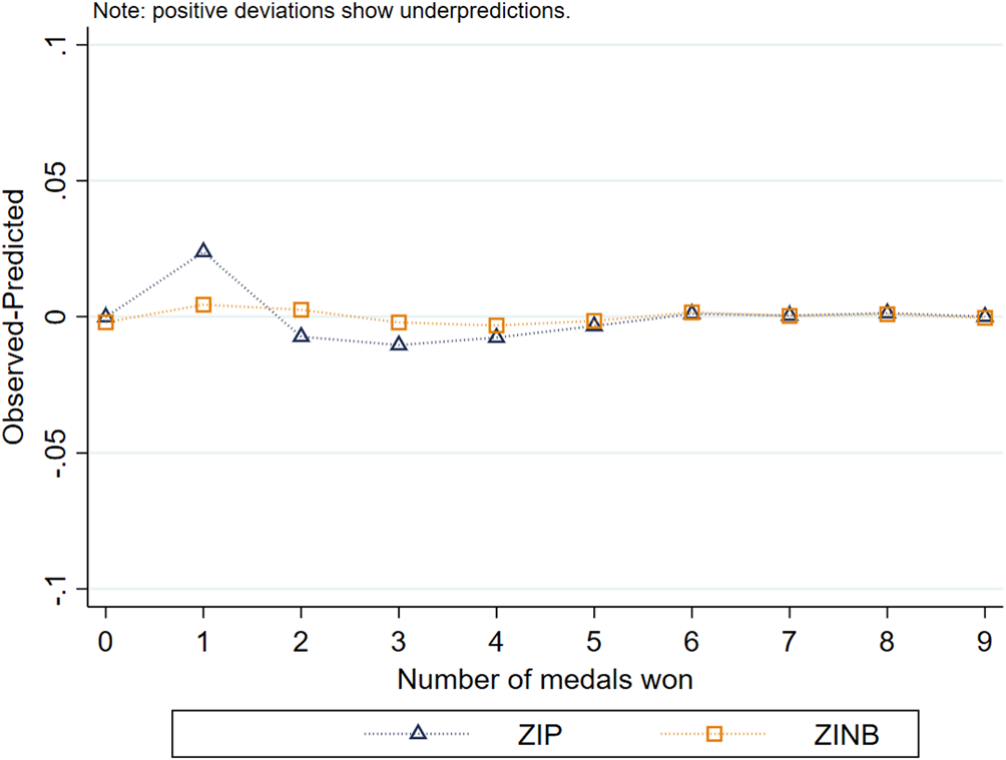
Fitting of ZINB and ZIP models.

The likelihood-ratio test showed α > 0 in the models with total medal count, justifying the decision to use ZINB in favour of ZIP. We present the fitting of the two models in Fig. 3. We estimated the test illustrated in this Figure with the model defined in Eq. (2) but the results of the other models showed similar patterns (other test results are available on request). The Voung test was significant at the 1% level; the use of zero-inflated regression is a better choice than the standard negative binomial model.

### Robustness analysis

We have also performed robustness tests to ensure the reliability of our results. First, we examined how our results change by omitting the sport-level fixed effect (Table A2) with the same estimator (ZINB). Second, we used a different estimator also without sport-level fixed effects (Table A3). In the literature, the Tobit regression is often used for Olympic data as it can handle censored data well. In this case, however, the zero values are estimated differently from the zero-inflated negative binomial regression we use. The results of the robustness analysis are presented in the Supplementary Appendices.

Omitting the sport-level fixed effect from the analysis does not change the results remarkably. In some cases, the level of significance and the magnitude of the coefficients changed, but the same countries won more medals at the hosted Olympic Games when the sport-level fixed effect was included. With the Tobit estimator, we get slightly different results. About the same number of countries show a significant host effect as with the ZINB regression. Greece in this case was able to significantly increase its medal count at the Athens Olympics. However, when including control variables, most of the significant effects disappear. Australia and Great Britain are also the only two countries where the host effect is most detectable. The results suggest that our results are robust to both model specifications and estimators.

## Discussion

We investigated whether the host countries of the Olympic Games have benefitted from hosting in terms of medal surplus. The baseline models show that most host countries won significantly more medals compared to their usual medal counts. When we ran the estimations separately by gender, the results are rather mixed.

In the extended model with GDP per capita, population and communist bloc dummy, the results provide less support for the existence of the host effect. A significantly greater number of medals was only observed for two hosts when all independent variables were incorporated. Moreover, there was only two case of a significant host effect by gender when the models were estimated with the control variables. In all models, the Olympic Games in Sydney and London represented the biggest successes in terms of a medal surplus.

Our results reveal that a significant medals surplus is more pronounced for men than for women. Our results can partly confirm^12,17,23^ and partly refute the conclusions of previous studies^5^. We assume that host countries are already inherently more successful at men’s than women’s events. An increase in resources may have a greater effect on pre-existing infrastructure and professional knowledge. Another possible explanation could be gender differences in cultures. In countries where religious restrictions mean that women have fewer opportunities to participate in sports, they win fewer Olympic medals^14^. Also, gender cultural differences exist even in industrialised countries such as Japan and the USA^31^. These differences can also manifest themselves at the level of sport, causing a difference in the host effect. A further possible explanation could be that the host countries in the sample are successful in other sports in terms of gender. Since in some sports the host effect is not detectable^10^, it is possible that the different results can only be explained by this fact. Nevertheless, this discrepancy is a very important finding that requires further analysis for deeper understanding.

Another important finding is that already successful countries with a good socioeconomic background do not significantly increase their medal count when GDP per capita and population are controlled for. Consequently, if countries with similar characteristics are bidding for the Olympics, the narrative of medal surplus should be treated with caution in attempts to justify hosting.

The ex-ante effect of hosting is detectable in three cases, in Spain and Great Britain in female events, and in Brazil in male events. We cannot confirm the findings of previous papers that identified he existence of ex-ante effects^12,18,25^. We can only detect the ex-ante effect in three countries where a systematic process of strategic elite sport development presumably preceded the domestic event.

The post effects of previous host countries are more visible compared to the ex-ante ones. As Vagenas and Vlachokyriakou indicated, the presence of a mid-term effect exists which arises from the reduced cost and better conditions for Olympic preparation thanks to prior home staging. This mid-term effect was detected in four of seven cases. Spain, United States, Great Britain, and Brazil were able to maintain and increase their success after the hosting. In addition, almost all the significant results of Spain, Great Britain, and Brazil remained with the extended model. The London Olympic Games was undoubtedly the most rewarding hosting as the British athletes could increase the average performance not only on the host event but also on the former and the latter Olympics. None of the other countries in the sample present similar success. By the Tokyo Olympics, the effect had disappeared, but this decline might be due to the Covid-19 pandemic.

One of the key findings of the research concerns the previous host country that was identified with no significant pre, post and host effects. Greece was the only that was not associated with any medal surplus, and it has one of the worst economic situation in terms of GDP per capita among the countries in the sample. Hosting a mega-event like the Olympic Games may have a longstanding negative impact on the Olympic performance of less wealthy countries due to the huge financial burden of hosting. Agenda 2020 was introduced by the (IOC) partly to help avoid such failures that undermine the credibility of the Olympic movement. Nevertheless, the next Summer Olympics, already awarded in line with Agenda 2020, will not be in such danger: France, the United States, and Australia seem like safe choices.

We find that the medal surplus associated with the host effect is rarely detectable. This finding in line with the results of Singleton et al. They find that the magnitude of effect decreases, the recent host countries are not able to win as many additional medals as the formerly Olympic hosts. In our research, we investigate whether the Olympic Games do lead significant medal gains, and show that this can only be proven in some cases. Based on our findings, recommend that bidding countries should be cautious about relying on the medal gains from hosting the Olympic Games in the future.

Another explanation for our results is the sport-level data and the host effect in Olympic sports. In some sports, especially where performance is measured objectively and there is no judging, the host effect does not exist^10^. The countries in the sample we used were probably typically good in these sports. For example, athletes from the United States and Australia tend to do well in swimming, or athletes from the United States, Great Britain, and China in athletics, where the host effect does not present.

We must emphasize the limitation of our study due to the sample selection. We used only data about the last seven Summer Olympic Games but the other Olympic Games could be also analysed using our methodology. Consequently, our results only apply to this sample, we do not have information on the Summer Olympics before 1996. The study has some natural extensions. First, our approach can be applied for the full sample, and specifically to the Winter Olympics. The difference between the male and female host effects is another possible line of research. None of the previous studies have addressed this issue in depth, while we have provided evidence of a more significant host effect in male events.

## Supplementary Information

Supplementary Tables.

## Data Availability

The data that support the findings of this study are available on request from the corresponding author. The data are not publicly available due to commercial restrictions.
