# Supplementary material for: The less obvious effect of hosting the Olympics on sporting performance
Source: Sci Rep. 2023 Feb 2;13:819. doi: 10.1038/s41598-022-27259-8 (PMC9895060; doi:10.1038/s41598-022-27259-8)
Supplement: Supplementary file 1 — Supplementary Tables. [file 41598_2022_27259_MOESM1_ESM.docx]

# Appendices

Table A1. Summary statistics of variables.

| Variable | N | Mean | SD | Min | Max |
| --- | --- | --- | --- | --- | --- |
| Medal Total | 13421 | 0.497 | 1.551 | 0 | 33 |
| Men Medal Total | 13421 | 0.272 | 0.914 | 0 | 19 |
| Women Meda Total | 13421 | 0.207 | 0.814 | 0 | 18 |
| Host | 13421 | 0.021 | 0.145 | 0 | 1 |
| OG96 | 13421 | 0.003 | 0.052 | 0 | 1 |
| OG00 | 13421 | 0.003 | 0.055 | 0 | 1 |
| OG04 | 13421 | 0.003 | 0.053 | 0 | 1 |
| OG08 | 13421 | 0.003 | 0.056 | 0 | 1 |
| OG12 | 13421 | 0.003 | 0.054 | 0 | 1 |
| OG16 | 13421 | 0.003 | 0.056 | 0 | 1 |
| OG20 | 13421 | 0.004 | 0.061 | 0 | 1 |
| Pre96 | 13421 | 0.002 | 0.048 | 0 | 1 |
| Pre00 | 13421 | 0.002 | 0.042 | 0 | 1 |
| Pre04 | 13421 | 0.003 | 0.051 | 0 | 1 |
| Pre08 | 13421 | 0.002 | 0.047 | 0 | 1 |
| Pre12 | 13421 | 0.002 | 0.05 | 0 | 1 |
| Pre16 | 13421 | 0.003 | 0.054 | 0 | 1 |
| Pre20 | 13421 | 0.003 | 0.057 | 0 | 1 |
| Post96 | 13421 | 0.002 | 0.045 | 0 | 1 |
| Post00 | 13421 | 0.003 | 0.052 | 0 | 1 |
| Post04 | 13421 | 0.003 | 0.053 | 0 | 1 |
| Post08 | 13421 | 0.002 | 0.044 | 0 | 1 |
| Post12 | 13421 | 0.003 | 0.05 | 0 | 1 |
| Post16 | 13421 | 0.002 | 0.05 | 0 | 1 |
| Post20 | 13421 | 0.003 | 0.052 | 0 | 1 |
| lnGDPpc | 12957 | 9.236 | 1.345 | 5.223 | 12.192 |
| lnPOP | 13201 | 16.535 | 1.91 | 9.195 | 21.056 |
| Communist bloc | 13421 | 0.25 | 0.433 | 0 | 1 |
| AM (total medal) | 13421 | 0.497 | 1.398 | 0 | 30.143 |
| AM (men medal) | 13421 | 0.272 | 0.784 | 0 | 15.571 |
| AM (women medal) | 13421 | 0.207 | 0.689 | 0 | 14.571 |
| Year | 13421 |  |  | 1,996 | 2,020 |

Table A2. Effects of hosting the Olympic Games by host without sport-level fixed effect.

|  | Baseline model | | | Extended model | | | |
| --- | --- | --- | --- | --- | --- | --- | --- |
|  | Total | Men | Women | | Total | Men | Women |
|  | (1) | (2) | (3) | | (4) | (5) | (6) |
| main |  |  |  | |  |  |  |
| OG96 | 0.513^***^ | 0.514^*^ | 0.252 | | 0.0702 | 0.202 | -0.333 |
|  | (0.186) | (0.270) | (0.360) | | (0.187) | (0.269) | (0.366) |
|  |  |  |  | |  |  |  |
| OG00 | 0.461^***^ | 0.237 | 0.686^***^ | | 0.342^**^ | 0.106 | 0.569^***^ |
|  | (0.176) | (0.266) | (0.216) | | (0.171) | (0.254) | (0.216) |
|  |  |  |  | |  |  |  |
| OG04 | 0.335 | 0.342 | 0.325 | | 0.435 | 0.364 | 0.529 |
|  | (0.268) | (0.278) | (0.409) | | (0.277) | (0.279) | (0.429) |
|  |  |  |  | |  |  |  |
| OG08 | 0.450^**^ | 0.189 | 0.575^**^ | | 0.116 | -0.0243 | 0.227 |
|  | (0.224) | (0.372) | (0.276) | | (0.213) | (0.360) | (0.260) |
|  |  |  |  | |  |  |  |
| OG12 | 0.672^***^ | 0.543^**^ | 0.548^**^ | | 0.508^***^ | 0.425^**^ | 0.372^*^ |
|  | (0.184) | (0.218) | (0.221) | | (0.175) | (0.209) | (0.218) |
|  |  |  |  | |  |  |  |
| OG16 | 0.264 | 0.716^**^ | -0.210 | | 0.332 | 0.798^***^ | -0.143 |
|  | (0.249) | (0.308) | (0.421) | | (0.250) | (0.310) | (0.431) |
|  |  |  |  | |  |  |  |
| OG20 | 0.417^**^ | 0.438^*^ | 0.239 | | 0.101 | 0.210 | -0.142 |
|  | (0.211) | (0.257) | (0.316) | | (0.205) | (0.251) | (0.298) |
|  |  |  |  | |  |  |  |
| AM | 0.762^***^ | 1.384^***^ | 1.431^***^ | | 0.680^***^ | 1.285^***^ | 1.306^***^ |
|  | (0.0281) | (0.0473) | (0.0719) | | (0.0263) | (0.0481) | (0.0604) |
|  |  |  |  | |  |  |  |
| lnGDPpc |  |  |  | | 0.338^***^ | 0.269^***^ | 0.403^***^ |
|  |  |  |  | | (0.0242) | (0.0240) | (0.0320) |
|  |  |  |  | |  |  |  |
| lnPOP |  |  |  | | 0.130^***^ | 0.0714^***^ | 0.183^***^ |
|  |  |  |  | | (0.0144) | (0.0175) | (0.0206) |
|  |  |  |  | |  |  |  |
| Communist bloc |  |  |  | | 0.610^***^ | 0.534^***^ | 0.620^***^ |
|  |  |  |  | | (0.0507) | (0.0589) | (0.0734) |
|  |  |  |  | |  |  |  |
| OG FE | YES | YES | YES | | YES | YES | YES |
|  |  |  |  | |  |  |  |
| Sport FE | NO | NO | NO | | NO | NO | NO |
|  |  |  |  | |  |  |  |
| Constant | -1.582^***^ | -2.223^***^ | -2.557^***^ | | -7.102^***^ | -6.046^***^ | -9.673^***^ |
|  | (0.0643) | (0.0736) | (0.106) | | (0.393) | (0.418) | (0.547) |
| inflate |  |  |  | |  |  |  |
| lnGDPpc | -1.218^***^ | -1.275^***^ | -1.278^***^ | | -0.234 | 0.446 | -1.096^**^ |
|  | (0.120) | (0.229) | (0.177) | | (0.648) | (1.057) | (0.448) |
|  |  |  |  | |  |  |  |
| lnPOP | -1.211^***^ | -1.563^***^ | -1.287^***^ | | -1.331^***^ | -1.570^***^ | -1.423^***^ |
|  | (0.102) | (0.232) | (0.138) | | (0.142) | (0.225) | (0.285) |
|  |  |  |  | |  |  |  |
| Constant | 27.88^***^ | 32.36^***^ | 30.43^***^ | | 19.05^**^ | 15.15 | 28.70^***^ |
|  | (2.120) | (4.353) | (3.097) | | (7.551) | (9.807) | (7.703) |
| Observations | 12957 | 12957 | 12957 | | 12957 | 12957 | 12957 |

Robust standard errors in parentheses

^*^ *p* < 0.1, ^**^ *p* < 0.05, ^***^ *p* < 0.01

Table A3. Effects of hosting the Olympic Games estimated with Tobit regression

|  | Baseline model | | | Extended model | | |
| --- | --- | --- | --- | --- | --- | --- |
|  | Total | Men | Women | Total | Men | Women |
|  | (1) | (2) | (3) | (4) | (5) | (6) |
| main |  |  |  |  |  |  |
| OG96 | 0.290 | 0.287 | -0.260 | -0.0606 | 0.131 | -0.568 |
|  | (0.321) | (0.319) | (0.377) | (0.318) | (0.332) | (0.370) |
|  |  |  |  |  |  |  |
| OG00 | 1.080^***^ | 0.771^**^ | 0.886^**^ | 0.856^***^ | 0.737^**^ | 0.710^**^ |
|  | (0.328) | (0.309) | (0.359) | (0.322) | (0.330) | (0.349) |
|  |  |  |  |  |  |  |
| OG04 | 0.796^**^ | 0.478 | 0.786^*^ | 0.714^*^ | 0.571 | 0.714 |
|  | (0.387) | (0.350) | (0.470) | (0.379) | (0.381) | (0.458) |
|  |  |  |  |  |  |  |
| OG08 | 1.375^***^ | 0.689^**^ | 1.174^***^ | 0.830^***^ | 0.391 | 0.715^**^ |
|  | (0.306) | (0.303) | (0.317) | (0.304) | (0.323) | (0.312) |
|  |  |  |  |  |  |  |
| OG12 | 1.239^***^ | 1.014^***^ | 0.727^*^ | 0.983^***^ | 0.862^***^ | 0.460 |
|  | (0.323) | (0.302) | (0.379) | (0.317) | (0.321) | (0.367) |
|  |  |  |  |  |  |  |
| OG16 | 0.401 | 0.720^**^ | -0.229 | 0.240 | 0.678^*^ | -0.416 |
|  | (0.372) | (0.321) | (0.505) | (0.363) | (0.350) | (0.487) |
|  |  |  |  |  |  |  |
| OG20 | 0.854^***^ | 0.574^*^ | 0.703^**^ | 0.435 | 0.404 | 0.232 |
|  | (0.309) | (0.294) | (0.339) | (0.301) | (0.317) | (0.328) |
|  |  |  |  |  |  |  |
| AM | 1.392^***^ | 1.615^***^ | 1.886^***^ | 1.301^***^ | 1.622^***^ | 1.697^***^ |
|  | (0.0150) | (0.0211) | (0.0373) | (0.0153) | (0.0261) | (0.0346) |
|  |  |  |  |  |  |  |
| lnGDPpc |  |  |  | 0.372^***^ | 0.267^***^ | 0.392^***^ |
|  |  |  |  | (0.0256) | (0.0268) | (0.0339) |
|  |  |  |  |  |  |  |
| lnPOP |  |  |  | 0.202^***^ | 0.133^***^ | 0.251^***^ |
|  |  |  |  | (0.0168) | (0.0176) | (0.0220) |
|  |  |  |  |  |  |  |
| Communist bloc |  |  |  | 0.726^***^ | 0.618^***^ | 0.644^***^ |
|  |  |  |  | (0.0686) | (0.0722) | (0.0876) |
|  |  |  |  |  |  |  |
| OG FE | YES | YES | YES | YES | YES | YES |
|  |  |  |  |  |  |  |
| Sport FE | NO | NO | NO | NO | NO | NO |
|  |  |  |  |  |  |  |
| Constant | -2.335^***^ | -2.022^***^ | -3.239^***^ | -9.132^***^ | -7.121^***^ | -11.05^***^ |
|  | (0.0796) | (0.0705) | (0.118) | (0.419) | (0.433) | (0.578) |
| / |  |  |  |  |  |  |
| sigma_u | 0.808^***^ | 3.73e-08^***^ | 0.930^***^ | 0.647^***^ | 0.696^***^ | 0.765^***^ |
|  | (0.0406) | (9.21e-10) | (0.0519) | (0.0407) | (0.0426) | (0.0499) |
|  |  |  |  |  |  |  |
| sigma_e | 1.649^***^ | 1.512^***^ | 1.573^***^ | 1.655^***^ | 1.488^***^ | 1.580^***^ |
|  | (0.0232) | (0.0269) | (0.0317) | (0.0236) | (0.0265) | (0.0322) |
| Observations | 13421 | 13421 | 13421 | 12957 | 12957 | 12957 |

Robust standard errors in parentheses

^*^ *p* < 0.1, ^**^ *p* < 0.05, ^***^ *p* < 0.01
